# Effects of Cautery-Assisted Palatal Stiffening With and Without Uvulectomy on Quality of Life in Patients With Obstructive Sleep Apnea and Snoring: A Retrospective Cohort Study

**DOI:** 10.7759/cureus.77487

**Published:** 2025-01-15

**Authors:** Abdulrahman Alosaimi, Abdulrahman F Alzamil, Rahaf O Alharbi, Fatima K Bughamar, Ibrahim A Tawfiq, Waleed Janahi

**Affiliations:** 1 Department of Otolaryngology, Head and Neck Surgery, Ohud Hospital, Medina, SAU; 2 Department of Otolaryngology, Head and Neck Surgery, Bahrain Royal Medical Services, Riffa, BHR; 3 Department of Otolaryngology, Head and Neck Surgery, King Hamad University Hospital, Muharraq, BHR; 4 College of Medicine, Taibah University, Medina, SAU

**Keywords:** cautery-assisted palatal stiffening (caps), obstructive sleep apnea, quality of life, sleep surgery, snoring, uvulectomy

## Abstract

Introduction: Obstructive sleep apnea (OSA) and snoring are common conditions that disrupt sleep, leading to significant physical and emotional challenges that affect daily life and overall well-being. While non-surgical treatments like continuous positive airway pressure (CPAP) are effective, many patients find them challenging to maintain. Cautery-assisted palatal stiffening (CAPS), with or without uvulectomy, offers a minimally invasive surgical alternative to improve symptoms and enhance quality of life (QoL).

Aim: This study aims to assess and compare the effects of CAPS, with and without uvulectomy, on post-surgical QoL in patients with OSA or snoring.

Materials and methods: This retrospective cohort study included 94 patients who underwent CAPS, with or without uvulectomy, at King Hamad University Hospital in Bahrain to treat OSA or snoring. QoL was assessed using the Short-Form Health Survey (SF-36), evaluating physical and emotional well-being.

Results: Among the 94 patients, CAPS improved physical functioning (mean score: 83.03) and general health (72.98). A trend toward greater emotional well-being was observed in the CAPS-only group compared to the uvulectomy group (p = 0.057). Over half of the participants (53.2%) reported feeling significantly better overall. CAPS effectively enhanced physical QoL, with some limitations remaining in emotional health.

Conclusions: CAPS and CAPS with uvulectomy significantly improve post-surgical QoL, especially in physical functioning, though some emotional challenges may persist. These findings support CAPS as a beneficial option for patients, particularly for enhancing physical well-being.

## Introduction

Obstructive sleep apnea (OSA) and snoring are common conditions that disrupt sleep and significantly impact patients' physical, mental, and emotional well-being. These disorders are associated with various health issues and can reduce health-related quality of life (QoL), often leading to daytime fatigue, cognitive impairment, and an increased risk of cardiovascular problems [1-5]. Management options range from lifestyle modifications and non-surgical treatments, such as continuous positive airway pressure (CPAP) devices, to surgical interventions. However, patient adherence to CPAP can be challenging due to discomfort, inconvenience, and the need for nightly use [6-8]. Thus, surgical options are often explored, particularly for patients who find non-surgical treatments ineffective or difficult to maintain.

Recently, cautery-assisted palatal stiffening (CAPS) has emerged as a minimally invasive alternative to traditional surgical options, such as uvulopalatopharyngoplasty (UPPP) and laser-assisted uvulopalatoplasty (LAUP) for stiffening the soft palate, reducing its vibration and, consequently, snoring [9]. CAPS is associated with lower complication rates and reduced recovery times compared to CPAP therapy and more invasive surgeries [10]. CAPS with uvulectomy may provide additional relief by further reducing airway obstruction, although the outcomes and benefits of combining these procedures remain an area of ongoing investigation.

These treatments offer potentially reliable management of snoring and mild OSA by removing the obstruction of airways at its origin, which is essential in enhancing QoL. The use of CAPS in recent times has increased significantly, necessitating research on the procedure's effectiveness with and without uvulectomy to optimize patient outcomes. Despite the increased clinical use, few comparative studies directly focus on the effects of these two approaches on QoL after surgery. In addition, CAPS is effective in reducing snore. However, the added benefits of performing uvulectomy have not been explored, especially regarding physical and emotional improvement in patients. Therefore, a comparative study on the efficacy of CAPS with uvulectomy and CAPS without uvulectomy would impact clinical practice and develop optimization strategies for QoL.

This study aims to retrospectively evaluate and compare the QoL outcomes of CAPS with and without uvulectomy. In addition, it will apply the Short-Form Health Survey (SF-36) to both the physical and emotional domains of QoL to provide evidence that may guide clinical decision-making in managing OSA and snoring.

## Materials and methods

Study design and settings

This retrospective cohort study evaluated the post-surgical QoL in patients treated with CAPS, with or without uvulectomy, at King Hamad University Hospital in Bahrain. This study was approved by the Institutional Review Board, with IRB number 22-501. In both CAPS with and without uvulectomy, a cautery-assisted technique was used to achieve the desired outcomes. For CAPS without uvulectomy, the soft palate was carefully stiffened under local anesthesia with sedation by creating controlled scarring to reduce vibration and airway obstruction. In CAPS with uvulectomy, the uvula was gently removed under local or general anesthesia, depending on the patient's preference, with special care taken to minimize tissue damage. Hemostasis was ensured in both procedures using bipolar cautery. Patients were closely monitored after surgery for any complications, such as bleeding or swallowing difficulties, and were provided with clear recovery guidelines, including pain relief strategies and dietary advice to support their healing process.

Participants

Adult patients (≤18 years) diagnosed with OSA or snoring and treated with CAPS with or without uvulectomy at King Hamad University Hospital in Bahrain between January 2017 and August 2024 were included. This timeframe was chosen because CAPS began to be used more prominently in clinical practice at the hospital around 2017. Patients were excluded if they had incomplete follow-up data or significant comorbidities that might independently affect QoL outcomes. Key comorbidities considered were hypertension, diabetes, cardiovascular disease, and chronic respiratory conditions, as these conditions are strongly associated with OSA severity and may impact both treatment efficacy and recovery.

Data collection

The SF-36, which evaluates physical and emotional health domains, was used to assess QoL [11]. The SF-36 survey was administered online to all eligible participants through a secure link, allowing for a standardized measure of post-surgical outcomes. Data were collected retrospectively from patient records spanning the study period from January 2017 to August 2024.

Statistical analysis

Data analysis was conducted using Statistical Package for the Social Sciences (IBM SPSS Statistics for Windows, IBM Corp., Version 27.0.1, Armonk, NY). Descriptive statistics summarized demographic and clinical characteristics, while the Mann-Whitney U test was used to compare SF-36 scores between the CAPS-only and CAPS with uvulectomy groups. Spearman's rank correlation was applied to assess associations between age and QoL domains. A p-value < 0.05 was considered statistically significant.

## Results

Demographics and CAPS with uvulectomy and without uvulectomy distribution among study participants

The study included 94 participants with a mean age of 45.19 ± 11.38 years. The majority of the participants were male (N = 80, 85.1%), while females comprised a smaller portion (N = 14, 14.9%). Seventy-four participants (78.7%) had CAPS without uvulectomy, whereas 20 (21.3%) underwent CAPS with uvulectomy (Table 1).

**Table 1:** Demographics and CAPS with and without uvulectomy distribution among study participants CAPS: Cautery-assisted palatal stiffening

		Mean	SD
Age		45.19	11.38
		N	%
Gender	Female	14	14.9
	Male	80	85.1
Uvulectomy	No	74	78.7
	Yes	20	21.3

Summary of QoL dimensions (SF-36) among study participants

The QoL dimensions assessed using the SF-36 among study participants showed that the highest scores were observed in physical functioning with a mean of 83.03 ± 24.74 and a median of 95.00 (IQR: 70.00-100.00).

Self-Reported Health Status and Impact of Health on Daily Activities Among Patients Post-CAPS With Uvulectomy and CAPS Without Uvulectomy

**Figure 1:**
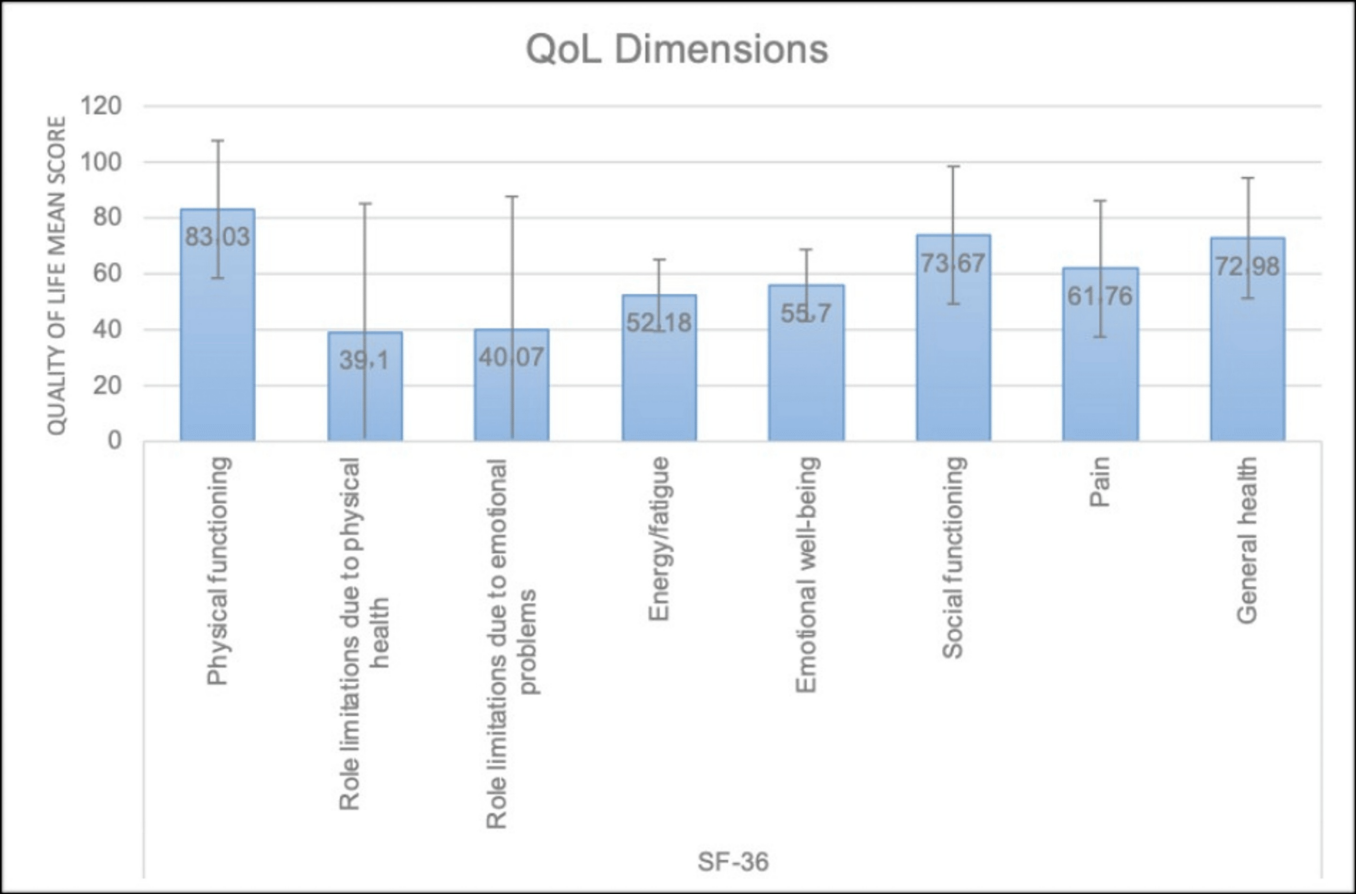
Mean scores with error bars for SF-36 QoL dimensions SF-36: Short-Form Health Survey; QoL: Quality of life

When asked about their general health, 42 (44.7%) patients rated it as excellent, while 25 (26.6%) considered it very good, 14 (14.9%) good, seven (7.4%) acceptable, and six (6.4%) bad. Compared to their preoperative condition, 50 (53.2%) felt much better, 20 (21.3%) a little better, and 19 (20.2%) noted no change, while three (3.2%) and two (2.1%) reported much worse and slightly worse health, respectively. Regarding daily activities, 51 (54.3%) patients reported no impact on extreme activities, while 25 (26.6%) were affected a little and 18 (19.1%) a lot. Medium-intensity activities showed similar trends, with 61 (64.9%) patients experiencing no limitation, 21 (22.3%) a little, and 12 (12.8%) a lot. Activities like shopping and climbing stairs mostly showed no significant impact. Health-related limitations in activities such as walking, bathing, and climbing stairs were minimal, with over 70% reporting no significant restriction. Regarding work performance over the past four weeks, 59 (62.8%) and 58 (61.7%) patients had reduced completion time and achieved less than desired, respectively. Emotional health also played a role, with 54 (57.4%) patients reporting decreased working time. For 47 (50%) of respondents, social interactions remained unaffected, while 17 (18.1%) experienced moderate interference. Pain levels varied, with 34 (36.2%) patients reporting very light pain and 23 (24.5%) light pain, while a small portion faced severe or very intense pain. The influence of physical health on emotions included feelings of boredom (N = 31, 33%) and lack of motivation (N = 34, 36.2%), while 29 (30.9%) patients reported feeling energetic. Only seven (7.4%) patients frequently felt tired, and 37 (39.4%) experienced frequent frustration or depression. Health and psychological conditions significantly affected personal relationships for 22 (23.4%) patients, while 51 (54.3%) reported no impact. Lastly, only four (4.3%) patients often felt sick, while 36 (38.3%) described their health as excellent, and 65 (69.1%) disagreed with the notion that their health was worsening. Role limitations due to physical health had a mean of 39.10 ± 45.87 and a median of 0.00 (IQR: 0.00-100.00), while role limitations due to emotional problems had similar scores with a mean of 40.07 ± 47.54 and a median of 0.00 (IQR: 0.00-100.00). Energy/fatigue reported a mean of 52.18 ± 13.05 and a median of 50.00 (IQR: 50.00-60.00), and emotional well-being had a mean of 55.70 ± 12.75 with a median of 60.00 (IQR: 48.00-60.00). Social functioning presented a mean of 73.67 ± 24.63 and a median of 75.00 (IQR: 62.50-100.00). Pain levels had a mean of 61.76 ± 24.60 with a median of 66.25 (IQR: 45.00-90.00). Lastly, general health had a mean score of 72.98 ± 21.57 and a median of 80.00 (IQR: 60.00-90.00) (Figure 1 and Tables 2-8).

**Table 2:** Summary of quality-of-life dimensions (SF-36) among study participants SF-36: Short-Form Health Survey

Quality of Life Metric	Mean	SD	Median	IQR
Physical functioning	83.03	24.74	95.00	70.00-100.00
Role limitations due to physical health	39.10	45.87	0.00	0.00-100.00
Role limitations due to emotional problems	40.07	47.54	0.00	0.00-100.00
Energy/fatigue	52.18	13.05	50.00	50.00-60.00
Emotional well-being	55.70	12.75	60.00	48.00-60.00
Social functioning	73.67	24.63	75.00	62.50-100.00
Pain	61.76	24.60	66.25	45.00-90.00
General health	72.98	21.57	80.00	60.00-90.00

**Table 3:** Self-reported health status and impact of health on daily activities among patients post-CAPS with and without uvulectomy (Questions 1 and 2) CAPS: Cautery-assisted palatal stiffening

		N	%
1. How do you describe your health condition in general?	Acceptable	7	7.4
	Bad	6	6.4
	Excellent	42	44.7
	Good	14	14.9
	Very good	25	26.6
2. Compared to before the operation, how do you evaluate your health condition now in general?	A little better	20	21.3
	A little worse	2	2.1
	Much better	50	53.2
	Much worse	3	3.2
	There is no difference	19	20.2

**Table 4:** Self-reported health status and impact of health on daily activities among patients post-CAPS with and without uvulectomy (Question 3) CAPS: Cautery-assisted palatal stiffening

The following questions are about daily activities during your normal day.		A little		No, it does not		Yes, it does	
		N	%	N	%	N	%
3. Does your current health limit your daily activity?	Extreme activities	25	26.6	51	54.3	18	19.1
	Moderate activities	21	22.3	61	64.9	12	12.8
	Carrying groceries	15	16.0	69	73.4	10	10.6
	Climbing the stairs	15	16.0	65	69.1	14	14.9
	Climbing one ladder	4	4.3	86	91.5	4	4.3
	Kneeling or standing	12	12.8	74	78.7	8	8.5
	Walk 1 km	9	9.6	72	76.6	13	13.8
	100 steps	12	12.8	75	79.8	7	7.4
	Short walking	13	13.8	77	81.9	4	4.3
	Bathing	7	7.4	84	89.4	3	3.2

**Table 5:** Self-reported health status and impact of health on daily activities among patients post-CAPS with and without uvulectomy (Questions 4 and 5) CAPS: Cautery-assisted palatal stiffening

4. During the past four weeks, to what extent has your physical health affected your work or usual daily activities?	No		Yes	
	N	%	N	%
Completion time decreased	35	37.2	59	62.8
Accomplished less than you wanted to	36	38.3	58	61.7
I said my activities	38	40.4	56	59.6
Difficulty in activities	38	40.4	56	59.6
5. In the past four weeks, to what extent have emotional problems (such as feeling depressed or anxious) interfered with your ability to work or carry out usual daily activities (e.g., reduced working hours, lower productivity, or difficulty concentrating)?				
Working time decreased	40	42.6	54	57.4
Say achievement	38	40.4	56	59.6
I said activities	35	37.2	59	62.8

**Table 6:** Self-reported health status and impact of health on daily activities among patients post-CAPS with and without uvulectomy (Questions 6-8) CAPS: Cautery-assisted palatal stiffening

		N	%
6. After the procedure, to what extent did your physical health or emotional problems interfere with your social activities with family, friends, neighbors, or groups?	A little	20	21.3
	Extremely	2	2.1
	Moderate	17	18.1
	Never	47	50.0
	Quite	8	8.5
7. How much physical pain have you experienced over the past four weeks?	Light	23	24.5
	Moderate	21	22.3
	Severe	6	6.4
	Very intense	10	10.6
	Very light	34	36.2
8. How does the pain affect your usual work (including work outside the home and housework)?	A little	19	20.2
	Extremely	5	5.3
	Moderate	27	28.7
	Never	36	38.3
	Quite	7	7.4

**Table 7:** Self-reported health status and impact of health on daily activities among patients post-CAPS with and without uvulectomy (Questions 9 and 10) CAPS: Cautery-assisted palatal stiffening

9. What is the effect of the data below on your feelings?	Always		Most of the time		Some time		A little		No effect	
	N	%	N	%	N	%	N	%	N	%
Bored of work	7	7.4	11	11.7	15	16.0	30	31.9	31	33.0
Vital	8	8.5	16	17.0	21	22.3	20	21.3	29	30.9
Worried about your health	14	14.9	11	11.7	20	21.3	17	18.1	32	34.0
Not motivated to work	7	7.4	15	16.0	17	18.1	21	22.3	34	36.2
You have energy	16	17.0	19	20.2	21	22.3	15	16.0	23	24.5
Frustrated or depressed	8	8.5	8	8.5	14	14.9	27	28.7	37	39.4
Overwork	10	10.6	13	13.8	17	18.1	27	28.7	27	28.7
Happy	12	12.8	24	25.5	21	22.3	15	16.0	22	23.4
Tired	7	7.4	13	13.8	19	20.2	23	24.5	32	34.0
10. To what extent does your health and psychological condition affect your relationships, such as relatives, friends, and neighbors?	3	3.2	7	7.4	22	23.4	11	11.7	51	54.3

**Table 8:** Self-reported health status and impact of health on daily activities among patients post-CAPS with and without uvulectomy (Question 11) CAPS: Cautery-assisted palatal stiffening

	Always		Mostly		I don’t know		No, never	
11. How true are each of the following about your health condition?	N	%	N	%	N	%	N	%
I get sick a lot	4	4.3	13	13.8	19	20.2	58	61.7
My health is moderate	31	33.0	35	37.2	11	11.7	17	18.1
My health is getting worse	5	5.3	10	10.6	14	14.9	65	69.1
My health is excellent	32	34.0	36	38.3	13	13.8	13	13.8

Spearman’s rank correlation coefficients between age and SF-36 QoL dimensions

The Spearman's rank correlation analysis between age and SF-36 QoL dimensions among study participants revealed no significant correlations. The correlations were generally weak, with physical functioning showing an r of -0.111 (p = 0.287) and role limitations due to physical health having an r of -0.061 (p = 0.561). Role limitations due to emotional problems were minimally correlated (r = -0.019, p = 0.858). Positive correlations were noted for energy/fatigue (r = 0.143, p = 0.170), emotional well-being (r = 0.075, p = 0.474), social functioning (r = 0.154, p = 0.138), pain (r = 0.093, p = 0.371), and general health (r = 0.077, p = 0.461). However, none of these correlations reached statistical significance, indicating that age did not significantly impact the SF-36 QoL dimensions in this cohort (Table 9).

**Table 9:** Spearman's rank correlation coefficients between age and SF-36 QoL dimensions SF-36: Short-Form Health Survey; QoL: Quality of life

SF-36	r	Sig. (2-tailed)	N
Physical functioning	-0.111	0.287	94
Role limitations due to physical health	-0.061	0.561	94
Role limitations due to emotional problems	-0.019	0.858	94
Energy/fatigue	0.143	0.170	94
Emotional well-being	0.075	0.474	94
Social functioning	0.154	0.138	94
Pain	0.093	0.371	94
General health	0.077	0.461	94

Comparison of SF-36 QoL scores between CAPS with uvulectomy and CAPS without uvulectomy patients

**Figure 2:**
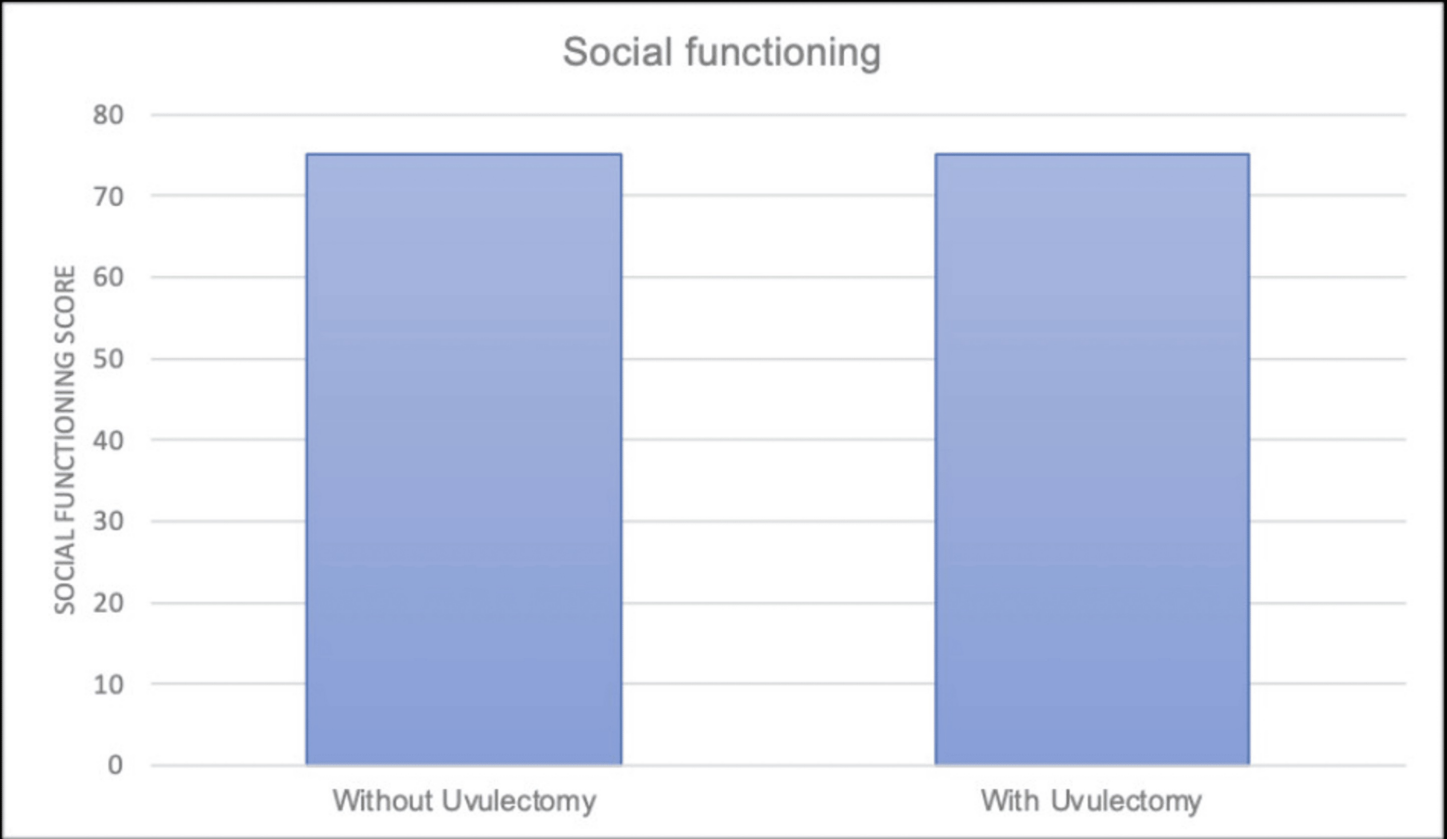
Comparison of social functioning scores between CAPS with and without uvulectomy patients CAPS: Cautery-assisted palatal stiffening

The Mann-Whitney U test revealed no significant differences between the two groups across most domains of the SF-36. Specifically, physical functioning, role limitations due to physical health, role limitations due to emotional problems, energy/fatigue, social functioning, pain, and general health scores showed p-values greater than 0.05, indicating no significant difference between CAPS with uvulectomy and CAPS without uvulectomy patients. However, emotional well-being approached border significance with a p-value of 0.057, suggesting a trend toward better scores in the CAPS without the uvulectomy group compared to the CAPS with the uvulectomy group. The median scores for both groups were generally similar across domains, except emotional well-being and general health, where the uvulectomy group reported slightly higher median scores (Table 10 and Figure 2).

**Table 10:** Comparison of SF-36 QoL scores between CAPS with and without uvulectomy patients CAPS: Cautery-assisted palatal stiffening; SF-36: Short-Form Health Survey; QoL: Quality of life

	Did you undergo uvula removal or uvula replacement?				
	Without uvulectomy		With uvulectomy		P-value
	Median	IQR	Median	IQR	
Physical functioning	95.00	75.00-100.00	92.50	67.50-100.00	0.649
Role limitations due to physical health	0.00	0.00-100.00	25.00	0.00-100.00	0.497
Role restrictions as a result of emotional problems	0.00	-100.00	0.00	0.00-100.00	0.950
Energy/fatigue	50.00	50.00-60.00	50.00	40.00-57.50	0.468
Emotional well-being	60.00	52.00-64.00	50.00	42.00-60.00	0.057
Social functioning	75.00	62.50-100.00	75.00	56.25-100.00	0.717
Pain	67.50	45.00-90.00	61.25	52.50-85.00	0.582
General health	80.00	60.00-90.00	67.50	55.00-80.00	0.069
^U ^Independent Samples Mann-Whitney U test *p<0.05, Significant					

## Discussion

This study explores the QoL of patients who have undergone CAPS without uvulectomy or CAPS with uvulectomy. The results demonstrate that both procedures favorably impact patients' QoL, particularly in physical functioning, general health, and social functioning. However, particular areas, like role limitations due to physical and emotional problems, showed more variable outcomes, indicating that while the procedures are beneficial, they may not fully resolve all aspects of patients' QoL.

This study's predominance of male participants is noteworthy, with 85.1% of the sample being male. This gender distribution aligns with previous research indicating that OSA, a common indication for these procedures, is more prevalent in males [12]. Additionally, the higher frequency of uvuloplasty over uvulectomy observed in this study reflects current clinical preferences, as uvuloplasty is often considered less invasive and associated with a quicker recovery time [13].

Regarding the QoL outcomes, the high scores observed in the physical functioning domain suggest that both CAPS without uvulectomy and CAPS with uvulectomy significantly improve patients' ability to engage in physical activities. This is consistent with other studies that have reported improvements in daytime functioning and reduced symptoms related to OSA following these procedures [14,15]. Techniques like radiofrequency ablation and ultrasound surgical devices are also used for snoring and mild OSA, offering precise tissue removal and reduced complications. However, CAPS and CAPS with uvulectomy are simpler, more cost-effective, and have shorter recovery times. Comparative studies are needed to evaluate their relative benefits. This study highlights the effectiveness of CAPS and uvulectomy in improving QoL. CAPS reduces soft palate vibration, while uvulectomy enhances airway patency, offering a minimally invasive alternative for managing snoring and mild OSA [16,17].

In this study, the lower scores in role limitations due to physical health (mean = 39.10 out of 100) and emotional problems (mean = 40.07 out of 100) highlight that some patients continue to experience limitations post-operatively. This could be due to the residual or recurrent symptoms of OSA, which may not be entirely alleviated by surgery alone.

Energy/fatigue and emotional well-being scores also presented areas of concern. The mean energy/fatigue score of 52.18 suggests that while there is some improvement, a significant portion of patients may still experience fatigue post-operatively. This aligns with studies that indicate surgical interventions for OSA may not fully restore standard sleep architecture, leading to continued fatigue [18]. Emotional well-being, with a mean score of 55.70, indicates moderate improvements, but not to the extent seen in physical domains. While abnormal size and shape of the uvula are often perceived as a primary cause of snoring, these may more frequently be consequences of chronic snoring rather than its root cause. Addressing this misconception through patient education could alleviate emotional distress and enhance therapeutic strategies, ultimately improving emotional well-being [19].

The self-reported health status and the impact of health on daily activities provide further context for understanding patients' experiences post-surgery. Notably, over half of the participants (53.2%) reported feeling much better post-operatively. However, the fact that 20.2% reported no change and a small percentage (3.2%) felt worse suggests that individual outcomes can vary significantly, potentially due to the severity of preoperative symptoms, surgical technique, or post-operative care. This variability highlights the importance of individualized patient assessments and setting realistic expectations before surgery [20].

There was no significant relationship between age and QoL outcomes, suggesting a comparative efficacy of CAPS without uvulectomy and CAPS with uvulectomy across different age groups. However, this contrasts with previous findings suggesting that age may influence surgical outcomes due to the differences in tissue elasticity and its capacity for healing at age [21]. This inconsistency could be caused by various study limitations, including the relatively small sample size and peculiarities within the characteristics of the population studied.

Similarly, there was an insignificant difference in the QoL scores of CAPS without uvulectomy versus CAPS with uvulectomy. However, regarding the emotional well-being scores, the trend appeared for the uvuloplasty patients to have better scores, suggesting potential for the less invasive procedure. This was consistent with research indicating that the less invasive a surgical procedure is, the less post-operative pain there is, which often means a faster recovery, and the less it can affect one's emotional psyche [22].

On the other hand, CAPS without uvulectomy and CAPS with uvulectomy showed relatively light post-operative pain, which is essential for patient satisfaction and recovery. However, a minority of the patients showed severe pain, emphasizing the significance of post-operative care to optimize outcomes [23,24]. The degree and type of pain relief were not directly assessed in this study but were indirectly evaluated through the SF-36 survey. Most patients reported light to very light pain post-operatively, with minimal reports of severe pain. Future research could explore pain relief in greater depth for comparison with other studies.

Limitations

This study had various limitations, including the small sample size of 94 participants with predominantly male participation, limiting the generalizability of findings to a larger population. In addition, self-reported measures of QoL and health status may be biased since participants' perceptions have subjective origins and may be influenced by other factors. This study evaluated QoL at a single post-operative time point, limiting insight into the timeline of improvement. Longitudinal studies are needed to track recovery and QoL changes over time. Additionally, weight parameters, such as BMI, were not assessed, which may play a critical role in understanding the severity of OSA and surgical outcomes.

Future research

Future research should involve longitudinal studies with longer follow-up durations to assess the long-term residual or improved living quality after CAPS without uvulectomy. In addition, larger sample sizes are essential with more diverse representation for better generalizability of results. Moreover, the variation in surgical techniques, the severity of preoperative symptoms, and the after-care should be investigated to establish these variables' influence on a patient's QoL. A more balanced gender distribution, consideration of age, comorbidities, and psychological support are also essential to provide a better insight into the factors affecting the QoL after these procedures.

## Conclusions

CAPS and CAPS with uvulectomy significantly improve post-surgical QoL, especially in physical functioning and general health. However, some patients had challenges in emotional well-being and would be considered to require further comprehensive care. These findings highlight the potential of CAPS as a viable treatment option for improving the OSA and snoring patient's physical QoL. Research should explore the long-term outcomes and emotional well-being to enhance therapeutic strategies.
